# Rapid implementation of ultraviolet germicidal irradiation and reuse processes for N95 respirators at a health system during the coronavirus disease 2019 (COVID-19) pandemic

**DOI:** 10.1017/ice.2020.1386

**Published:** 2020-12-16

**Authors:** Brian P. Zunner-Keating, Patricia De Jesus Alberto, Sarah J. Sweeney, Annabelle de St. Maurice, Erik Eggins, Shaunté C Walton, Martin Lingard, Yuhan Kao, Daniel Z. Uslan

**Affiliations:** 1 Performance Excellence, UCLA Health, Los Angeles, California; 2 Emerging Infectious Disease Preparedness, UCLA Health, Los Angeles, California; 3 Pediatric Infectious Diseases, David Geffen School of Medicine at UCLA, Los Angeles, California; 4 Office of Environmental Health & Safety, UCLA Health, Los Angeles, California; 5 Clinical Epidemiology & Infection Prevention, UCLA Health, Los Angeles, California; 6 Logistics, UCLA Health, Los Angeles, California; 7 Center for Nursing Excellence, UCLA Health, Los Angeles, California; 8 Division of Infectious Diseases, David Geffen School of Medicine at UCLA, Los Angeles, California

## Abstract

An N95 respirator ultraviolet germicidal irradiation and reuse program was rapidly implemented at an academic health system in the United States during the coronavirus disease 2019 pandemic. This process continues to be a safe and effective way to slow the consumption rate of N95 respirators.

With the emergence of coronavirus disease 2019 (COVID-19), health systems have been challenged with developing creative strategies to deal with N95 respirator supply chain disruptions coupled with increased demand. N95s are recommended by public health authorities for the care of patients with COVID-19 undergoing aerosol-generating procedures and for care involving other transmissible diseases such as measles and tuberculosis.^1,2^

From March through August 2020, UCLA Health admitted 565 confirmed COVID-19 inpatients. N95 respirator consumption increased dramatically from <9,000 per month prior to COVID-19 to >83,000 per month in March 2020. To ensure that healthcare workers would have an adequate supply of N95s, a multidisciplinary team was assembled to evaluate the feasibility and later implement ultraviolet germicidal irradiation (UVGI) and reuse processes for N95 respirators. These processes contributed to a decrease in the N95 consumption rate to <40,000 per month in June 2020 despite significant increases in COVID-19 admissions. Herein, we describe how these processes were developed and rapidly scaled to areas of high N95 use at UCLA Health.

## Methods

### Setting

The following processes were applied to >60 targeted clinical areas at UCLA Health including inpatient floors, emergency departments, operating rooms, and procedural clinics. These areas are all encompassed within or adjacent to 2 academic hospitals comprising >750 inpatient beds. UCLA Health guidelines indicate that surgical masks are to be used for routine care of suspected and confirmed COVID-19 patients, and N95 respirators are only used when performing aerosol-generating procedures. Extended use of N95s is not allowed, and reuse is only allowed following UVGI decontamination. UCLA Health offers safety goggles and face shields for care of suspected and confirmed COVID-19 patients; however, face shields are preferred when wearing an N95 to minimize contamination of the respirator.

### Equipment

These processes rely on ultraviolet (UV) machines called UVEnclosures (UV-Concepts, Inc., Littleton, CO) to irradiate N95 respirators. One machine was placed proximally to the primary COVID-19 intensive care unit in each hospital. A standard UV cycle time of 4 minutes was established to irradiate the respirators with 1,200 mJ/cm^2^ to ensure that all pathogens are inactivated.

Given the prolonged UV exposure, postirradiation quantitative fit testing was performed by UCLA Health’s Environmental Health and Safety department with various N95 models including 3M models 8210, 9210, 1860S (3M, St Paul, MN), Halyard model 46767 (Halyard Health, Alpharetta, GA), and Moldex model 1513 (Moldex, Culver City, CA). Each respirator was donned by a tester prior to UVGI, then again between each successive decontamination cycle, for up to 5 cycles. Quantitative respirator fit testing equipment was used between cycles and confirmed that each respirator maintained its seal, with a fit factor >100, throughout the testing.

### Process

The UCLA Health N95 collection and distribution processes were modeled after those published by Nebraska Medicine^3^ with several significant changes. Multiple publications support the safety and efficacy of UVGI for decontaminating respirators for reuse.^4–6^ However, UCLA chose to have respirators labeled prior to initial use and returned to the original wearer after processing due to concerns about optics and socialization of the process with employees.

Wherever possible, doffing stations are located immediately outside of patient rooms where the respirators are doffed then placed into a labeled brown paper bag. This location was chosen to minimize the risk of contamination from staff walking around with respirators or accidentally leaving bags containing a dirty respirator at a workstation. Staff who may wear N95 respirators are encouraged to avoid wearing makeup to decrease the likelihood of soiling. Because the respirators cannot be cleaned of visible contaminants, soiled respirators are discarded.

**Fig. 1.**
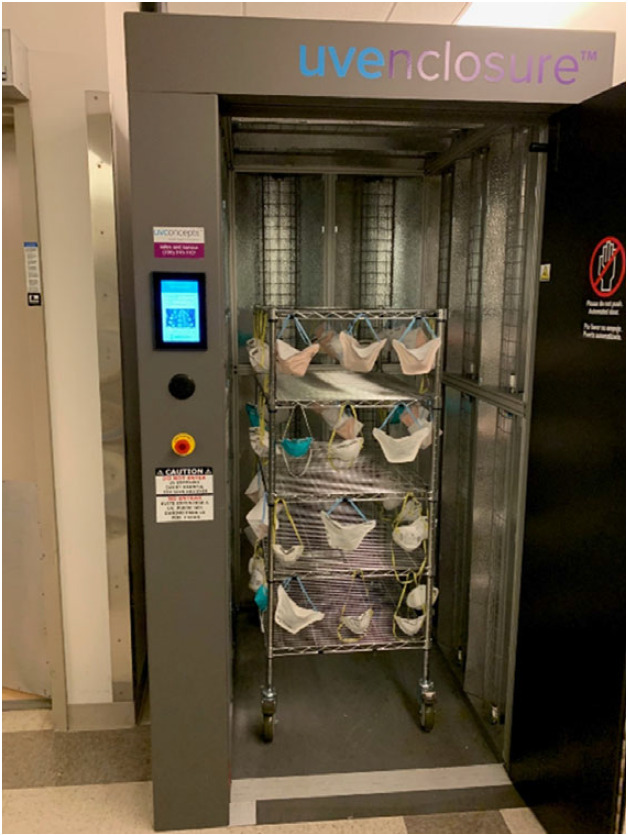
Respirators are suspended from a wire shelving unit, then rolled into the ultraviolet (UV) machine for ultraviolet germicidal irradiation (UVGI).

Couriers make rounds routinely to collect the paper bags. Continuous pickups, compared to daily pickups, enable the same respirator to be reused several times throughout a clinician’s shift. At the UV machine, the courier partners with the machine operator to decontaminate and repackage respirators (Figure 1). The courier then returns the irradiated respirators to the designated area for reuse.

To ensure that the respirator integrity is maintained throughout the process, staff must perform positive and negative pressure seal tests prior to each reuse. The seal test includes donning the respirator then inhaling and exhaling while ensuring that there is no airflow around the perimeter of the respirator and that the respirator puckers in slightly during inhale. These instructions are included in the training and standard operating procedure documents posted at the donning stations.

### Change management

This process was pilot tested on a COVID-19 unit then expanded to other clinical areas based on readiness and N95 consumption rate. Standard operating procedures for wearing and for irradiating the respirators were created to help staff follow the processes consistently. Frontline staff and leaders were engaged in process development and a town hall was held where the co-chief infection prevention officer and director of emerging infectious disease preparedness answered staff questions and concerns.

## Results

From March 31, 2020, through August 24, 2020, >80,000 N95 respirators underwent UVGI at UCLA Health, ranging from 321 to 1,006 daily. Furthermore, 61% of the respirators processed only went through 1 UVGI cycle, 20% were decontaminated twice, 8% were decontaminated 3 times, 6% were decontaminated 4 times, and 6% of respirators were decontaminated 5 times.

Initially, the entire process ran 24 hours daily, but the collection and reprocessing shifted to a 12-hour daily operation due to staffing challenges. Night-shift clinicians are expected to collect their used N95s for pick-up the next morning, and these are available for reuse the following shift. The turnaround time from dirty respirator pickup to decontaminated respirator drop-off averages ~60 minutes. The busiest areas require up to 8 pickups per shift, while others require only 1.

Conservatively, the team estimates having capacity to decontaminate ~500 respirators per hour per UV machine, far outpacing even the highest demand. With increased staffing, this capacity could likely be doubled. Although the process initially utilized 19–23 full-time equivalent (FTE) positions per site, the workflows were streamlined to require only 6.3 FTE per site, with 3 staff members working a single 12-hour shift daily.

Regarding employee safety, UCLA Health monitors all COVID-19 employee exposures. Based on the exposures identified to date, there have been no known exposures related to the UVGI process itself or the respirators decontaminated through the UVGI process.

## Discussion

Although the labor costs to support N95 collection, decontamination, and redistribution process are significant compared to the cost savings, it did not require any additional positions. Instead, float, per diem, or redeployed staff from closed clinics were utilized. The cost of the 2 UV machines was estimated to have been offset after 5 months of use given the increased cost of N95s during the supply shortage. Ultimately, however, no direct cost savings accrued due to labor costs.

UCLA Health still has significant opportunities to increase employee compliance with respirator reuse after UVGI. Only 39% of respirators underwent UVGI more than once. Some reasons for this low reuse rate include improper labeling of the respirators, staff unwillingness to reuse their respirators, and staff forgetting to retrieve their decontaminated respirators.

UVGI has proven to be a safe and effective process for UCLA Health to ensure that our clinicians do not find themselves in a situation where they must compromise their safety to care for COVID-19 patients. UCLA Health plans on continuing this N95 UVGI and reuse process until there is no longer a reasonable risk of an N95 shortage.
